# A role for p38 MAPK in the regulation of ciliary motion in a eukaryote

**DOI:** 10.1186/1471-2121-12-6

**Published:** 2011-01-26

**Authors:** Margarida Ressurreição, David Rollinson, Aidan M Emery, Anthony J Walker

**Affiliations:** 1 School of Life Sciences, Kingston University, Penrhyn Road, Kingston upon Thames, Surrey KT1 2EE, UK; 2 Wolfson Wellcome Biomedical Laboratories, Zoology Department, The Natural History Museum, Cromwell Road, London SW7 5BD, UK

## Abstract

**Background:**

Motile cilia are essential to the survival and reproduction of many eukaryotes; they are responsible for powering swimming of protists and small multicellular organisms and drive fluids across respiratory and reproductive surfaces in mammals. Although tremendous progress has been made to comprehend the biochemical basis of these complex evolutionarily-conserved organelles, few protein kinases have been reported to co-ordinate ciliary beat. Here we present evidence for p38 mitogen-activated protein kinase (p38 MAPK) playing a role in the ciliary beat of a multicellular eukaryote, the free-living miracidium stage of the platyhelminth parasite *Schistosoma mansoni*.

**Results:**

Fluorescence confocal microscopy revealed that non-motile miracidia trapped within eggs prior to hatching displayed phosphorylated (activated) p38 MAPK associated with their ciliated surface. In contrast, freshly-hatched, rapidly swimming, miracidia lacked phosphorylated p38 MAPK. Western blotting and immunocytochemistry demonstrated that treatment of miracidia with the p38 MAPK activator anisomycin resulted in a rapid, sustained, activation of p38 MAPK, which was primarily localized to the cilia associated with the ciliated epidermal plates, and the tegument. Freshly-hatched miracidia possessed swim velocities between 2.17 - 2.38 mm/s. Strikingly, anisomycin-mediated p38 MAPK activation rapidly attenuated swimming, reducing swim velocities by 55% after 15 min and 99% after 60 min. In contrast, SB 203580, a p38 MAPK inhibitor, increased swim velocity by up to 15% over this duration. Finally, by inhibiting swimming, p38 MAPK activation resulted in early release of ciliated epidermal plates from the miracidium thus accelerating development to the post-miracidium larval stage.

**Conclusions:**

This study supports a role for p38 MAPK in the regulation of ciliary-beat. Given the evolutionary conservation of signalling processes and cilia structure, we hypothesize that p38 MAPK may regulate ciliary beat and beat-frequency in a variety of eukaryotes.

## Background

Motile cilia are microscopic membrane-bound extensions of certain cells that are vital for the survival and reproduction of many eukaryotes. By beating in a regular pattern these evolutionarily-conserved organelles exert mechanical force; they thus play important roles in motility of small organisms and facilitate fluid movement across epithelial surfaces in complex multicellular animals. In addition to their role in fluid movement, motile cilia have recently been found to possess sensory functions in mammals, a feature previously thought to be restricted to non-motile 'primary' cilia [1,2]. Given that motile cilia are essential to physiology it is not surprising that defects in these organelles cause multiple human disorders [3-5]. Such ciliopathies include primary ciliary dyskinesia resulting in an inability to clear mucous and debris from airways [6], hydrocephalus caused by abnormal spinal fluid movement in the ventricles of the brain [7], and situs inversus (inversions of the normal left/right symmetry of organs) a consequence of altered nodal flow during embryogenesis [8,9]. Motile cilia (and flagella, which are essentially long motile cilia) are also essential for the completion of the life cycles of various parasites of humans and animals. For example, they power locomotion of schistosome larvae (miracidia) enabling host-finding and thus infection of the snail intermediate host [10], and are required for migration of trypanosomes between gut and salivary glands in the tsetse fly vector [11], and for viability of the bloodstream trypanosome form [12].

Motile cilia (and flagella) are composed of nine microtubular doublets and, usually, two central mictrotubular singlets, comprising the axoneme; dynein arms and radial spokes associated with the axoneme generate and control axonemal bending and thus force generation. The regulation of ciliary beating has been the focus of much research [13], and proteomic studies including those on the model organism *Chlamydomonas reinhardtii*, have aimed to describe the repertoire of proteins present within eukaryotic cilia and flagella, or their component fractions [14-19]. The proteomic analysis of *Chlamydomonas *flagella revealed over 90 putative signal transduction proteins including kinases and phosphatases [14], some of which might be anchored to the axoneme [20], highlighting the importance of signalling and reversible protein phosphorylation in the function of motile cilia and flagella. This is further supported by the recent identification in this organelle of 32 flagella phosphoproteins with 126 *in vivo *phosphorylation sites [21]. Despite these advances, our understanding of kinase-mediated cell signalling mechanisms regulating ciliary motion is still rudimentary, being largely restricted to the roles of the cAMP-dependent protein kinase, protein kinase A (PKA) [13,22-24], and protein kinase C (PKC) [13,25-27].

The surface of the schistosome miracidium is almost entirely covered with numerous motile locomotory cilia which, in *Schistosoma mansoni*, emerge from 21 ciliated epidermal plates arranged in tiers [10]. The *S. mansoni *miracidium is fully developed in the egg when it is released in the faeces of the infected definitive (human) host; upon water contact the miracidium hatches and swims rapidly to locate a suitable intermediate freshwater snail-host. Miracidia movements respond rapidly to various environmental cues such as the presence of host-snail components [28] or salinity [29], suggesting that these motile cilia might possess sensory functions as is the case with *Paramecium *[reviewed in [2]]. While studying kinase-mediated cell signalling in *S. mansoni *during miracidia development we observed an unexpected event, namely that activation of p38 mitogen-activated protein kinase (p38 MAPK) attenuated miracidial swimming. This kinase, an orthologue of the yeast HOG kinase, participates in signalling cascades that regulate transcriptional responses to stress [30] as well as having other non-transcription factor targets such as cytosolic phospholipase A_2 _[31]. Here we report findings that support a role for p38 MAPK in the regulation of ciliary motion of the multicellular eukaryote *S. mansoni*.

## Results and Discussion

### Characterization of *S. mansoni* p38 MAPK

**Figure 1:**
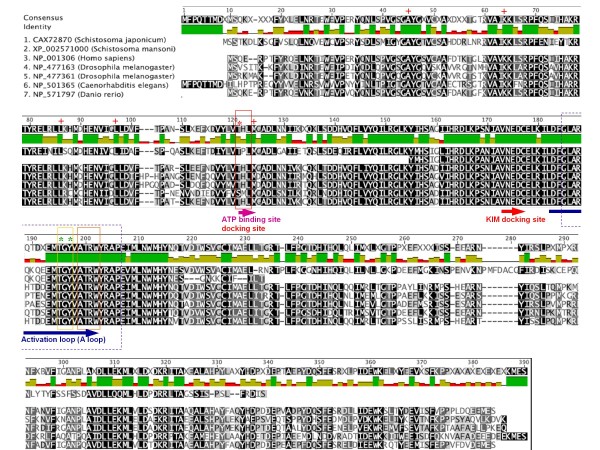
**Sequence alignment and domain analysis of *S. mansoni *p38 MAPK**. The *S. mansoni *putative p38 MAPK sequence (XP_002571000) was aligned with those for *S. japonicum *MAPK14a, *Homo sapiens *(p38 MAPKα, also known as MAPK14 isoform 1), *Drosophila melanogaster *(p38a MAPK and p38b MAPK), *Caenorhabditis elegans *(p38 MAPK family member, PMK1) and *Danio rerio *(MAPK 14a); accession numbers are shown. Multiple alignments were achieved using Geneious Pro 4.85 with Blosum62 cost matrix and default settings; shading of residues: black = 100% similar, dark grey = 80-100% similar, light grey = 60-80% similar and white < 60% similar. The ATP binding site, kinase interacting motif (KIM) docking site, and activation loop are indicated by coloured arrows. Within the activation loop, the conserved Thr-Gly-Tyr (TGY) phosphorylation motif is highlighted by the yellow box, with the phosphorylated residues (Thr and Tyr) central to kinase activation indicated with green asterisks; the substrate binding site is shown by the orange box. Sequence highlighted by the blue box is that used to generate the monoclonal anti-phospho p38 MAPK antibodies. The residues within the ATP binding site that are known to confer specificity and sensitivity of SB 230580 towards p38 MAPK are highlighted by the red box with the residue (Thr) most responsible for p38 MAPK inhibition indicated by the red asterisk; red crosses denote other known interaction sites between SB 203580 and p38 MAPK (see text for further details).

As with other MAPKs, p38 MAPK has been highly conserved during metazoan evolution [32]. Recently, the draft genomes for *S. mansoni *and *Schistosoma japonicum *were published [33,34] allowing *S. mansoni *p38 MAPK gene candidates to be identified and further assessed for similarity to p38 MAPKs from other organisms, including closely related schistosomes (Figure 1). For *S. mansoni *only a single putative p38 MAPK was identified and only partial cDNA reads were found; moreover, only one p38 MAPK was found in *S. japonicum *(Figure 1). Thus, in contrast to *D. melanogaster *and human which possess two and four p38 MAPKs respectively [32,35,36], it seems that schistosomes may possess one p38 MAPK orthologue only. Based on the partial sequence data (XP_002571000) spanning 74 amino acids, and supported by more complete data from *S. japonicum*, the identified *S. mansoni *p38 MAPK was most similar to p38α MAPK (MAPK 14) of humans, the most evolutionarily-conserved p38 MAPK [35]. In addition, there exist a number of putative exons on two separate scaffolds (Smp_scaff000038 and Smp_scaff06141) additional to the 74 amino acid fragment that, when translated, also match closely with the *S. japonicum *sequence; thus, we assume given the phylogenetic proximity between the two species that *S. mansoni *p38 MAPK is very similar to *S. japonicum *p38 MAPK. As for all other p38 MAPKs in the p38 subfamily, the dual phosphorylation site in the *S. mansoni *p38 MAPK activation loop reads Thr-Gly-Tyr (TGY)(Figure 1). The substrate binding site Ala-Thr-Arg-Trp (ATRW) is also conserved, as is the kinase interaction motif (KIM) docking site (Figure 1) which binds linear KIM sequences present in substrates and MAPK phosphatases [37]. Pair wise comparisons of the *S. mansoni *p38 MAPK fragment with corresponding sequences for other organisms revealed ~69-70% similarity with human, *Drosophila melanogaster*, *Caenorhabditis elegans*, or *Danio rerio*, and 86.5% with *S. japonicum*.

**Figure 2:**
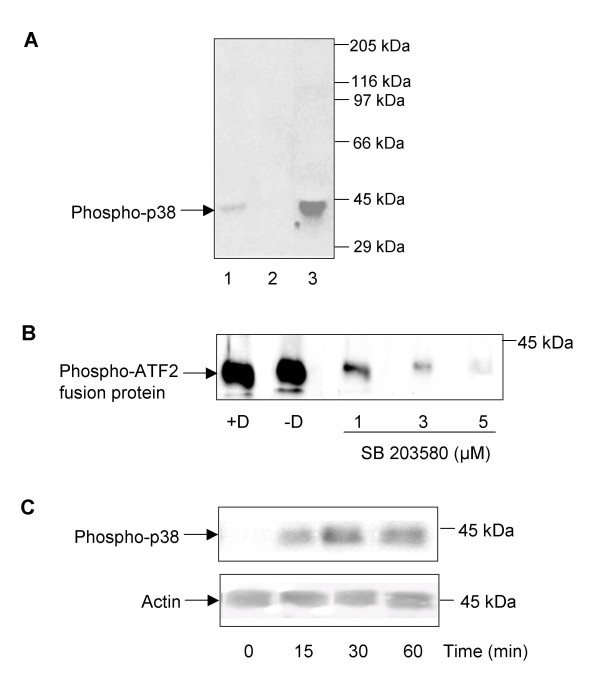
**Biochemical characterization of *S. mansoni *p38 MAPK**. (A) Immunodetection of phosphorylated *S. mansoni *p38 MAPK. Protein from astrocytoma (U251 MG) cells (lane 1), 900 freshly-hatched swimming miracidia (lane 2), or an adult worm pair (lane 3) was processed for western blotting using anti-phospho p38 MAPK antibodies. (B) Activated *S. mansoni *p38 MAPK phosphorylates activating transcription factor-2 (ATF-2), and SB 203580 inhibits p38 MAPK activity. p38 MAPK from adult worm pairs was immunoprecipitated using immobilized anti-phospho p38 MAPK antibodies and the immunoprecipitated protein used in an *in vitro *kinase assay to phosphorylate ATF-2 either in the presence of SB 203580 (1-5 μM) or DMSO (D+), or with neither (D-). Phosphorylation of ATF-2 by immunoprecipitated p38 MAPK was assessed by western blotting using anti-phospho ATF-2 antibodies. (C) Anisomycin activates p38 MAPK in *S. mansoni *miracidia. Freshly-hatched miracidia were exposed to anisomycin (20 μM) for various durations and phosphorylation of p38 MAPK in miracidia detected by western blotting with anti-phospho p38 MAPK antibodies; blots were also probed with anti-actin antibodies to demonstrate equal protein loading between lanes. Results shown in (A-C) represent those obtained in at least two independent experiments.

Anti-phospho p38 MAPK monoclonal antibodies were used in an attempt to detect phosphorylated p38 MAPK in *S. mansoni*. These antibodies, that bind p38 MAPK only when dually phosphorylated on Thr/Tyr of the TGY motif, have been used to detect phosphorylated p38 MAPK in many multicellular eukaryotes including *C. elegans *[38] and *D. melanogaster *[39]. Because phosphorylation on these residues results in activation of the enzyme, immunoreactivity directly correlates with p38 MAPK activity. The p38 MAPK amino acid sequence surrounding the TGY motif to which these antibodies are raised is highly-conserved between both *S. mansoni *and *S. japonicum*, and between *S. mansoni *and *C. elegans*, *D. melanogaster*, *D. rerio *and human (Figure 1). Western blotting of adult worm homogenates revealed that anti-phospho p38 MAPK antibodies recognized a single protein band with apparent molecular weight of approximately 42 kDa, essentially co-migrating with phosphorylated p38 MAPK from human astrocytoma (U251 MG) cells (Figure 2A). In marked contrast to adult worms, freshly-hatched swimming miracidia did not possess detectable levels of phosphorylated p38 MAPK (Figure 2A). A second antibody (anti-p38 MAPK) that detects p38 MAPK in vertebrates irrespective of phosphorylation state did not react with the *S. mansoni *protein precluding its use in this study.

A p38 MAPK immunoprecipitation kinase assay kit was next employed to confirm that the *S. mansoni *protein recognized by the anti-phospho p38 MAPK antibodies possessed p38 MAPK activity. Immunoprecipitates from adult worm homogenates phosphorylated the p38 MAPK substrate activating transcription factor 2 (ATF-2) (Figure 2B). The anti-inflammatory pyridinylimidazole compounds (SmithKline Beecham, SB) inhibit p38 MAPK with both activity of the phosphorylated enzyme and its autophosphorylation affected [40]. These inhibitors compete with ATP at the ATP binding site and exhibit no, or very weak inhibitory activity toward the closely related MAPKs, c-Jun N-terminal kinase (JNK) and extracellular signal-regulated kinase (ERK) [41]. SB 203580 inhibits both p38α and p38β MAPK and the interactions between the inhibitor and human kinases have been mapped using crystallography and amino acid substitution experiments [42,43]. The crucial residues thought to be involved in this interaction are shown in Figure 1; although falling outside the *S. mansoni *sequence fragment, these residues are conserved in *S. japonicum *p38 MAPK. We thus postulated that SB 203580 would inhibit *S. mansoni *p38 MAPK. Pre-incubation of immunoprecipitates with SB 203580 prior to kinase assay reduced ATF-2 phosphorylation considerably when compared to immunoprecipitates incubated in dimethyl sulfoxide (DMSO) vehicle alone; inhibition appeared dose dependent with 5 μM SB 203580 almost completely attenuating ATF-2 phosphorylation (Figure 2B). Thus the antibody recognizes an *S. mansoni *protein with p38 MAPK-like activity that can be inhibited by the p38 MAPK inhibitor SB 203580.

Anisomycin is a known potent stimulator of p38 MAPK phosphorylation, and therefore activation, so we considered that this compound might activate p38 MAPK in freshly-hatched swimming miracidia that lacked the phosphorylated (activated) form of the enzyme (Figure 2A). Western blotting revealed that treatment of these miracidia with 20 μM anisomycin for 15 min induced considerable p38 MAPK phosphorylation when compared to controls, which appeared to increase after 30 min and was sustained over 60 min (Figure 2C).

The results of the above biochemical experiments, coupled with the bioinformatic analysis, are commensurate with the *S. mansoni *immunoreactive protein being a p38 MAPK orthologue.

### Activated p38 MAPK is associated with *S. mansoni* cilia

**Figure 3:**
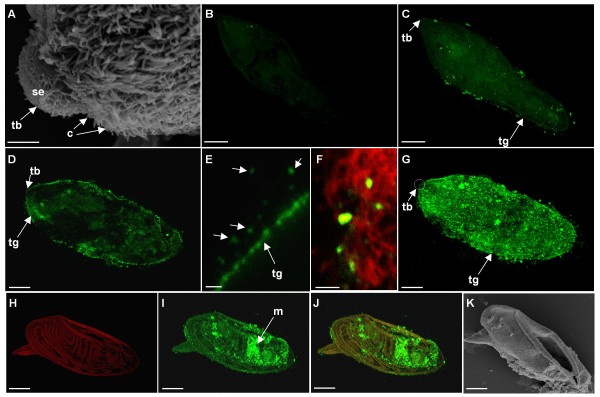
**Activated p38 MAPK localizes to cilia in *S. mansoni *miracidia**. (A) Scanning electron micrograph of the anterior of a freshly-hatched miracidium showing numerous cilia (c), and sensory endings (se) associated with the semi-spherical terebratorium (tb). (B - J) Immunolocalization of activated p38 MAPK (green) in *S. mansoni *miracidia following fixing and staining of parasites with anti-phospho p38 MAPK primary antibodies. (B) Freshly-hatched miracidium incubated without primary antibodies but with secondary antibodies alone (negative control). (C) Freshly-hatched swimming miracidium displaying weak patchy staining only localized adjacent to the tegument (tg) in areas corresponding to the cilia. (D) Serial optical z-section of a miracidium treated with 20 μM anisomycin for 30 min showing p38 MAPK activity in the regions occupied by cilia. (E and F) High power, optically zoomed, z-sections through discrete regions of cilia/tegument to capture the miracidium surface in one plane with (E) showing p38 MAPK localized to the shafts of the cilia (arrowed) and regions proximal to the tegument, and (F) showing co-localization with cilia (red) detected using anti-acetylated tubulin antibodies. (G) Miracidium treated with 20 μM anisomycin for 30 min for direct comparison with (C). (H) Autofluorescence (red) of *S. mansoni *egg containing (I, and J overlay) miracidium (m) with p38 MAPK activity in regions occupied by cilia. (K) Scanning electron micrograph of *S. mansoni *egg revealing common position of rupture during hatching, and correlating to rupture in H-J. Z-axis projections are shown in maximum pixel brightness mode. Bars: A, E and F = 2 μm; B-D, and G = 15 μm; H-K = 25 μm.

To determine the localization of activated p38 MAPK within *S. mansoni *miracidia, anti-phospho p38 MAPK antibodies and confocal laser scanning microscopy were used. Z-axis projections viewed in maximum pixel brightness mode revealed that freshly-hatched swimming miracidia possessed low levels of phosphorylated p38 MAPK, which is in agreement with western blotting analysis (Figures 2A and 2C). However where activation was evident, it appeared to be associated with the region occupied by the cilia (Figure 3C). Miracidia incubated with secondary antibodies alone possessed only background fluorescence (Figure 3B). When freshly-hatched swimming miracidia were incubated with 20 μM anisomycin for 30 min, a striking increase in p38 MAPK activation was observed (Figure 3G) whereas DMSO exposed (control) miracidia appeared similar to untreated miracidia (data not shown). Analysis of serial optical z-sections revealed activation largely at, or adjacent to, the ciliated surface of the parasite (e.g. Figure 3D). Fine z-sectioning and optical zooming revealed that activated p38 MAPK was associated with the shaft of the cilia (Figure 3E), further demonstrated through co-localization using anti-acetylated tubulin antibodies that permit visualization of these structures (Figure 3F). Immunoreactivity was present up to 6-7 μm from the tegument surface which correlates with the length (7-8 μm) of the cilia reported from transmission electron microscopy studies [10]. Staining for activated p38 MAPK was also observed in the tegument (Figures 3E); here the enzyme could either be associated with the base of the cilia, the microvilli (1 μm long [10]) that are known to exist among the cilia, the rootlet (2 μm long [10]) that is thought to provide support [44], or other structures. The semi-spherical terebratorium (anterior papilla) lacks cilia (Figure 3A) but possess filopodia-like sensory endings [10] thought to play a role in sensing the intermediate snail host. Although staining was observed at the terebratorium, it was noticeably less than that observed in the adjacent ciliated plates (Figure 3G).

While observing miracidia by immunofluorescence microscopy a solitary egg was found on the microscope slide, this egg had ruptured to allow hatching but fortunately still contained the miracidium which stained successfully with antibodies. Strikingly, and in contrast to swimming miracidia, the hatching miracidium possessed considerable p38 MAPK activity that *via *z-section analysis was found to be cilia-associated. Experiments were then designed in an attempt to re-capture this event and on several occasions miracidia were observed within ruptured eggs; such miracidia contained cilia-associated p38 MAPK activity considerably greater than that of freshly-hatched swimming miracidia (Figures 3H-K).

Although various kinases including PKA, casein kinase, adenylate kinase, cGMP-dependent protein kinase and a putative MAPK have been identified in proteomic screens of cilia/flagella e.g. [14,15], to our knowledge p38 MAPK has not been detected by such methods. P38 MAPK has however recently been localized to the post-acrosomal region and upper flagellum mid-piece of human sperm by fluorescence microscopy [45]. Although sperm tails are classified as flagella, their regulation differs from motile cilia in some aspects; sperm also possess a number of unique accessory structures [19] making them somewhat distinct from other motile cilia.

### Activation of p38 MAPK correlates with attenuation of cilia beat and swim velocity

**Figure 4:**
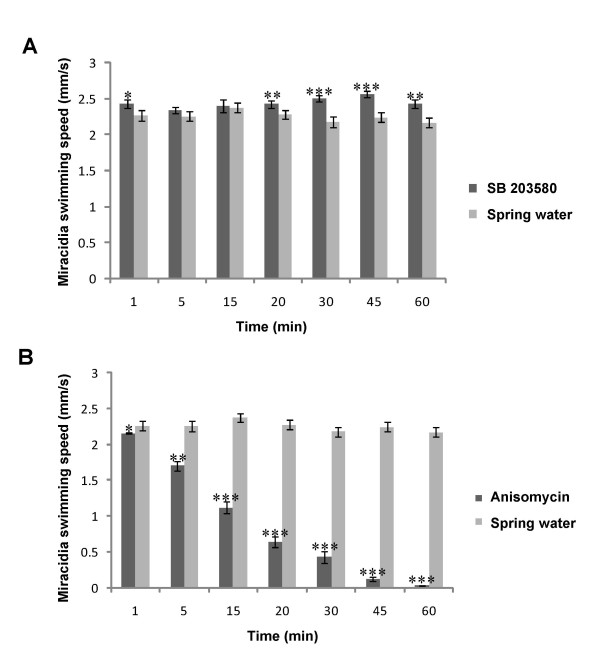
**p38 MAPK activation and inhibition affects *S. mansoni* miracidia swim velocity**. Freshly-hatched swimming miracidia were collected and incubated with (A) SB 203580 (1 μM) or (B) anisomycin (20 μM) in spring water, or spring water alone, and the effects on miracidia swim behaviour over 60 min captured by digital video microscopy. Calculation of swim velocities (mm/s) for 30 miracidia for each treatment and each time point was achieved using ImageJ and mean swim speeds (± SEM) were calculated. *P ≤ 0.05, **P ≤ 0.01, and ***P ≤ 0.001.

Because p38 MAPK was active in stationary miracidia within eggs but inactive in swimming miracidia, and because p38 MAPK localized to cilia, we next explored whether pharmacological activation or inhibition of p38 MAPK phenotypically affected ciliary function by performing swim velocity analyses. Miracidia were exposed to anisomycin or SB 203580 and their swim behaviour recorded by digital video microscopy (see additional file 1: Supplementary movie file). Determination of swim velocities revealed that freshly-hatched miracidia swam at speeds between 2.17 and 2.38 mm/s over 60 min (Figure 4A and B); DMSO treatment did not affect swim velocities over this duration (data not shown). However, anisomycin (20 μM) significantly attenuated miracidia swim velocity over time when compared to untreated (spring water) controls (*P *≤ 0.001) (Figure 4B); after 5 min and 30 min mean swim velocity was reduced by 25% to 1.70 mm/s (*P *≤ 0.01), and by 80% to 0.43 mm/s (*P *≤ 0.001), respectively. After 60 min anisomycin treatment swimming essentially ceased. On the other hand, SB 203580 (1 μM) significantly accelerated miracidia swim velocity (*P *≤ 0.001), particularly after 30 and 45 min treatment when it increased ~15 % (*P *≤ 0.001). Importantly, following 60 min anisomycin treatment, when spring water (to dilute the anisomycin concentration 10-fold) or SB 203580 (1 μM) were added, the miracidia started swimming displaying swim velocities of between 0.4 and 0.6 mm/s after 20 min (data not shown; see additional file 1: Supplementary movie file). That the effects of anisomycin could be reversed demonstrates that cessation of swimming was not due to larval death. Thus, the contrasting effects of SB 203580 and anisomycin on swim velocity and the finding that p38 MAPK localizes to the cilia are consistent with p38 MAPK playing a role in ciliary beat and thus miracidia swimming. Anisomycin is thought to indirectly activate p38 MAPK *via *cdc42/rac, MAPKKKs, MAPKK3 (MKK3) and MKK6 which ultimately phosphorylate p38 MAPK [46]. Importantly, orthologues of these pathway components have been identified in *S. mansoni *[33] suggesting that the mechanisms by which anisomycin exerts its effect on p38 MAPK are conserved between schistosomes and higher eukaryotes. P38 MAPK phosphatases have also been identified in *S. mansoni *[33], allowing rapid dephosphorylation of p38 MAPK when required. Although aspects of sperm flagella make them distinct from other motile cilia it is interesting that p38 MAPK inhibition by SB 203580 was recently shown to stimulate forward and hyperactivated motility of human sperm [45].

The mechanism by which p38 MAPK controls ciliary beat is not known. Active p38 MAPK could attenuate ciliary beat either by direct or indirect interactions involving phosphorylation of axonemal components or components within a ciliary signal transduction cascade, respectively. Studies with *C. reinhardtii *have demonstrated that phosphorylation and dephosphorylation control flagella motility [20] and have highlighted the complex nature of protein phosphorylation in this organelle [21]. Many potential kinase substrates exist that co-ordinate ciliary motility including the dyneins [47] and central pair kinesin KLP1 [48], but further characterisation of kinase substrates within cilia is needed.

Only a few other kinases have been reported to regulate cilia beat. PKA has been shown to attach to *Paramecium *ciliary axonemes, and strong evidence exists for PKA playing a positive role in *Paramecium *swimming and in controlling ciliary beat frequency of mammalian cilia [13,22-24]. Interestingly, PKA has also been implicated in regulating the ciliary motion of *S. mansoni *miracidia [49]. While cGMP-dependent protein kinase (cGMP) has also been shown to positively regulate ciliary beat frequency [13,50], like for p38 MAPK, PKC has been implicated in slowing ciliary beat [13,25-27]. Clearly, more research is needed into the influence of kinase activities on ciliary movement. For example, ERK1/2 has recently been found to bind radial spoke protein 3 in mammals and regulate its interaction with PKA [51]; however, the extent to which ERK actually influences motility *via *this interaction warrants investigation.

### Activation of p38 MAPK accelerates loss of cilia during larval transformation

**Figure 5:**
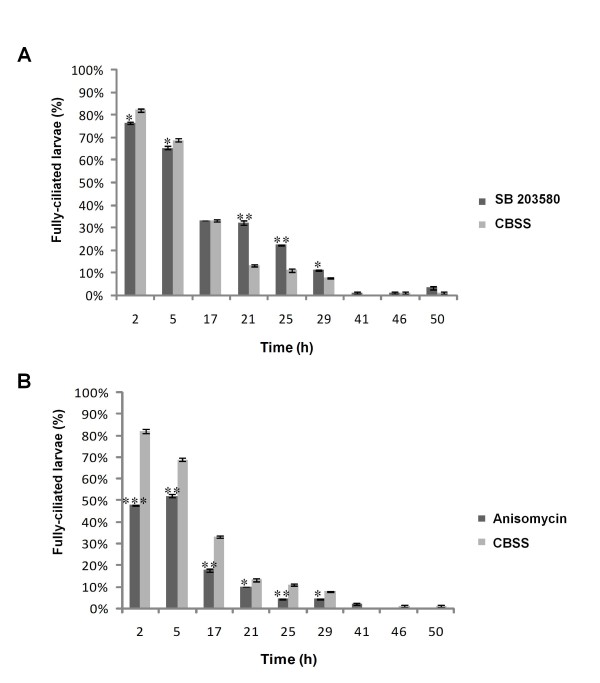
**p38 MAPK activation accelerates the liberation of ciliated plates in transforming *S. mansoni *larvae**. Cultures of larvae were exposed to (A) SB 203580 or (B) anisomycin, during transformation *in vitro *and the proportion of fully-ciliated parasites determined by microscopic examination over 50 h, compared to CBSS controls. Values shown are means (± SEM, n = 90 for each time point and each treatment from three independent experiments). *P ≤ 0.05, **P ≤ 0.01, and ***P ≤ 0.001.

When miracidia penetrate a suitable intermediate snail host they shed their ciliated plates and develop into the next larval stage, the mother sporocyst. This transformation can also be performed *in vitro *[52] during which miracidia stop swimming before the plates are released. As p38 MAPK activation attenuates miracidia swim speed whereas inhibition accelerates it we explored the effects of anisomycin and SB 203580 on deciliation and thus swimming during miracidium-to-mother sporocyst transformation *in vitro*. DMSO did not affect the deciliation rate when compared to Chernin's balanced salt solution (CBSS) controls and none of the treatments affected the survival of the developing larvae (data not shown). Anisomycin accelerated the shedding of ciliary plates considerably (Figure 5B); after only 2 h transformation 52 % of parasites had stopped swimming having shed at least some cilia in contrast to only 18 % of parasites in the CBSS control group (P ≤ 0.001; Figure 5B). At this time point 20 % of anisomycin-treated larvae had shed all their ciliated plates compared to none in CBSS alone (data not shown). This effect of anisomycin persisted throughout larval transformation (Figure 5B). Although the effects of SB 203580 were somewhat less marked, at 21 h, 25 h and 29 h significantly more miracidia were observed swimming (with all cilia attached) than were present in the CBSS control group (Figure 5A). Thus, p38 MAPK activation appears to accelerate the initial rate of *S. mansoni *miracidium transformation by attenuating cilia-mediated swimming behaviour leading to early release of ciliated plates.

## Conclusions

Here biochemical, innunohistochemical and functional data are presented that are consistent with p38 MAPK playing an important part in the regulation of ciliary beat and thus swimming behaviour of the multicellular eukaryote, *S. mansoni*. The marked difference in p38 MAPK activation between un-hatched or stationary miracidia and actively swimming miracidia is striking. Localization of active p38 MAPK to both the cilium shaft and the tegument of stationary miracidia implies that p38 MAPK might play multiple parts in co-ordinating swim behaviour, including sensory roles as described/hypothesized for motile cilia in other organisms including parasites [1,2,53]. Given the conservation of both signalling processes and structure/function of motile cilia, we hypothesize that p38 MAPK might regulate ciliary beat frequency in a variety of metazoans. Thus our findings could have implications for studies into motility of other important multicellular eukaryotes including parasites of humans, and for research into various human ciliopathies.

## Methods

### Sequence characterization of *S. mansoni* p38 MAPK

*S. mansoni *p38 MAPK gene candidates were identified from version 4.0 of the schistosome genome assembly by searching *S. mansoni *GeneDB hosted by the Wellcome Trust Sanger Institute, relying on the existing annotation [33,54]. Although only partial cDNA reads were found, they were further assessed for similarity to p38 MAPKs from other organisms using the NCBI tBLASTx search tool [55], limited to bilateria (organism) and the nucleotide dataset. Protein sequences of candidates with matches to p38 MAPK genes were aligned to those of other organisms, including that for *S. japonicum *[34] using Geneious Pro 4.85 (Biomatters Ltd, Auckland, New Zealand) with Blosum62 cost matrix, threshold = 1 and default parameters. Pair wise identity scores were also obtained using Geneious Pro.

### Isolation of *S. mansoni* miracidia and adult worms

Adult worms were recovered by portal perfusion of patent mice infected with *S. mansoni *(Belo Horizonte strain) and were immediately snap frozen in liquid nitrogen and stored at -80°C. Livers and spleens were then removed from the infected mice and *S. mansoni *eggs isolated; miracidia were then hatched from eggs for up to 2 h in natural spring water (Evian) and were collected under a dissecting microscope using a Pasteur pipette [52]. Miracidia were washed three times in spring water in a Stericup filter (0.45 μm PVDF membrane, Millipore, Watford, UK). The same filter was then used to concentrate the miracidia (to achieve approximately 10,000 miracidia/ml); enumeration of larvae was performed in aliquots under an inverted light microscope. Animal use received appropriate local ethical approval.

### Pharmacological activation and inhibition of p38 MAPK

The effect of the p38 MAPK activator anisomycin on p38 MAPK phosphorylation (activation) in *S. mansoni *was assessed by western blotting using anti-phospho p38 MAPK (Thr180/Tyr182) monoclonal antibodies (Cell Signalling Technology, New England Biolabs, Hitchin, UK) that recognize only the phosphorylated (activated) form of the enzyme. Freshly-hatched miracidia (~900 per treatment) were incubated in anisomycin (20 μM in spring water) or spring water containing vehicle (0.02% (v/v) DMSO) for varying durations (15, 30 or 60 min) and then immediately placed on ice and proteins extracted by adding an appropriate volume of 5x SDS-PAGE sample buffer followed by brief homogenization. Samples were then boiled for 5 min and sonicated briefly. After cooling, protease and phosphatase inhibitors (Sigma, Poole, UK) were added at the manufacturer's recommended concentrations and samples stored at -20°C prior to electrophoresis.

*Schistosoma mansoni *protein samples were separated on 10% SDS-PAGE gels and were transferred to nitrocellulose using a semi-dry electrotransfer unit (Bio-Rad, Hemel Hempsted, UK). After staining with Ponceau S to confirm homogeneous transfer, membranes were blocked for 1 h in 5% (w/v) non-fat dried milk, and then incubated anti-phospho p38 MAPK monoclonal antibodies (1/1000 in tris-buffered saline/0.1% Tween-20 (TTBS) containing 1% (w/v) BSA) overnight at 4°C. Next, blots were washed in TTBS and incubated for 2 h at room temperature in horse-radish peroxidase-congugated secondary antibodies (Cell signalling Technology; 1/5000 in TTBS) before further washing and incubation in West Pico chemiluminescent substrate (Pierce, Tattenhall UK) for 5 min. Immunoreactive bands were then visualised using cooled CCD GeneGnome chemiluminescence imaging system (Syngene, Cambridge, UK). Equal loading of proteins on blots was checked by stripping blots for 3 h at room temperature with Restore western blot stripping buffer (Pierce), before briefly washing blots in TTBS and incubating blots with anti-actin antibodies (1:3000 in TTBS; Sigma). Human astrocytoma (U251 MG) cell lysates, used as positive control for detection of phosphorylated p38 MAPK, were kindly provided by Suzanne Newton (Kingston University).

To determine p38 MAPK activities of proteins immunoprecipitated using anti-phospho p38 MAPK antibodies, a non-radioactive p38 MAPK activity assay kit was used (Cell Signalling Technology). Ten adult worm pairs were homogenized in cell lysis buffer (250 μl), the lysate cleared by centrifugation at 13,000 rpm in a microfuge for 10 min at 4°C, and the supernatant recovered. Subsequently, 2 μl of immobilized anti-phospho p38 MAPK (Thr180/Tyr182) monoclonal antibodies were added to the lysate and samples were gently mixed overnight at 4°C. Subsequently, the immune complex was washed twice in cell lysis buffer and re-suspended in kinase buffer before 1 μl of ATP and 1 μl of ATF-2 fusion protein were added to start the kinase reaction. After 30 min the reaction was terminated by adding an appropriate volume of 5x SDS-PAGE sample buffer. The samples were then boiled, sonicated and processed for western blotting using anti-phospho ATF-2 primary antibodies. All buffers and reagents used were provided in the p38 MAPK assay kit. In parallel experiments, the p38 MAPK inhibitor SB 203580 (1 μM, 2 μM, or 5 μM) or vehicle (0.02 % (v/v) DMSO) was added to the immunoprecipitates 15 min prior to the start of the kinase assay.

### Immunohistochemistry

Freshly-hatched swimming *S. mansoni *miracidia were either fixed immediately in absolute acetone or were treated with anisomycin (20 μM), or vehicle (0.02 % (v/v) DMSO), for 30 min prior to fixing. In some experiments, miracidia were left to hatch from eggs for short durations (10 - 20 min) prior to fixing in absolute acetone; this was done in an attempt to recover miracidia in the process of hatching from the egg. All parasites were then stored at 4 °C. For further preparation acetone was removed and samples washed twice with phosphate buffered saline (PBS) before being permeabilized in 0.3% (v/v) Triton X-100 for 1 h and washed with PBS prior to blocking in 10% (v/v) goat serum (Invitrogen, Paisley, UK) for 1 h. After a further wash with PBS, parasites were incubated with anti-phospho p38 MAPK mAb (1:50 in 5% (w/v) BSA) for 3 days on a microfuge tube rotator. The parasites were then washed twice in PBS for 1 h each and incubated in Alexa fluor 488 secondary antibodies (1:500 in 5% (w/v) BSA) for 24 h in the dark, followed by a further wash in PBS for 1 h. To detect cilia, miracidia were also incubated as above in anti-acetylated tubulin mouse monoclonal antibodies (1:100 in 5% (w/v) BSA; Sigma, Poole, UK) and Alexa fluor 594 secondary antibodies. Next, parasites were placed on microscope slides, left to air dry prior to mounting in Vectashield (Vecta Laboratories, Peterborough, UK) anti-bleaching medium, and sealed with transparent nail polish. All incubations were carried out at room temperature and incubations and washes were done in 2 ml screw cap tubes. Miracidia and eggs were then visualized on a Leica TCS SP2 AOBS confocal laser scanning microscope using a 20x dry objective or 40x and 63x oil immersion objectives and images collected with Leica software. Since *S. mansoni *miracidia autofluoresce, the signal received for the negative controls (i.e. those incubated only with secondary antibodies) was reduced. This was achieved by reducing the power level of the photomultiplier tube, which was then kept constant for all observations.

### Scanning Electron microscopy

For conventional scanning electron microscopy, acetone fixed miracidia or eggs were plated on individual coverslips and left to air dry, they were then placed in PBS for 4 h, dehydrated in ethanol, briefly soaked in hexamethyldisilazane and evaporated, and sputter-coated with gold-palladium. Specimens were then visualized on a Zeiss EVO50 scanning electron microscope.

### Analysis of *S. mansoni* swim velocity

Freshly-hatched miracidia in spring water were divided into 200 μl aliquots and exposed to either SB 203580 (1 μM), anisomycin (20 μM), vehicle (DMSO, 0.02% (v/v)), or were left untreated. Each sample was then immediately placed into a small sterile Petri dish and the 200 μl droplet spread out using a pipette; care was taken to ensure that the size and spread of the droplet was consistent between experiments to minimize artefacts in measurement owing to the miracidia swimming out of the horizontal plane during recordings. Light influences considerably miracidia swimming behaviour, so light intensity and positioning also remained constant for all experiments which were performed at 27°C. Miracidia were videoed over 60 min. There were approximately 10 miracidia in each sample and at least 30 miracidia per treatment were analysed in three independent experiments. Visualization was achieved using an Olympus SZ4045 binocular dissecting microscope and avi-format video recordings were made using a JVC TK-1481 composite colour video camera linked to Studio Launcher Plus for Windows software. Digital videos were subsequently processed using the freely-available analysis software ImageJ [56] to determine swim path length of individual miracidia in 5s permitting swim velocities (mm/s) to be calculated at various time points after treatment.

### Analysis of deciliation during larval transformation

Recovered eggs from schistosome-infected mice were hatched in spring water containing penicillin and streptomycin (100 units/ml each). Collected miracidia were then washed, and concentrated using Stericup filters, in sterile Chernin's balanced salt solution, pH 7.2, [57] containing glucose and trehalose and the same antibiotics (CBSS^+^). Approximately 1500 miracidia were placed onto individual wells of 6-well cell culture plates (Nunc, Loughborough, UK) and further 2 ml of either CBSS^+^, or CBSS^+ ^containing DMSO, SB 203580, or anisomycin (0.02% (v/v), 1 μM, and 20 μM final concentrations, respectively) added. The culture plates were then placed in a dark, humidified chamber in an incubator at 26°C. Three independent experiments were performed and media was not changed during larval development. At various time points during development (4h - 55 h), 30 parasites from each sample were randomly selected using an inverted microscope and the percentage of parasites retaining all of their ciliated plates was recorded. Larvae were determined as being alive if they displayed either swimming or contractile movements, or if flame-cell flickering was visible [52].

### Statistical analysis

Statistical analysis was performed using Minitab 15 Statistical Software; two sample t-tests or analysis of variance (ANOVA) were performed as appropriate.

## Abbreviations

ATF-2: activating transcription factor 2; CBSS: Chernin's balanced salt solution; DMSO: dimethyl sulfoxide; MAPK: mitogen-activated protein kinase; PBS: phosphate buffered saline; PKA: protein kinase A; PKC: protein kinase C; TGY: Thr-Gly-Tyr; TTBS: tween-tris buffered saline.

## Authors' contributions

MR designed and performed experiments and wrote the manuscript. DR supervised the project and modified the manuscript. AM conducted the bioinformatics (in conjunction with MR) and modified the manuscript. AW carried out the scanning electron microscopy, supervised the project, designed the experiments, and wrote the manuscript. All authors read and approved the final manuscript.

## Supplementary Material

Additional file 1 **Supplementary Movie File**. Combined example videos of miracidia in spring water (control) or SB 203580 (1 μM in spring water) for 60 min, anisomycin (20 μM in spring water) for 30 min or 60 min, or revived after anisomycin treatment (60 min anisomycin followed by 20 min in SB 203580 (1 μM)). Miracidia swim speed is increased slightly by SB203580 and is attenuated after 30 min anisomycin treatment; swimming stops after 60 min in anisomycin and is revived following subsequent incubation in SB203580. Click here for file
